# Supplementary material for: The recreational-to-habitual shift in psychostimulant use is an economic demand parameter that is unrelated to drug consumption levels (under normal and punishment conditions)
Source: bioRxiv. 2026 May 21:2026.05.19.726350. Preprint. [Version 1] doi: 10.64898/2026.05.19.726350 (PMC13228279; doi:10.64898/2026.05.19.726350)
Supplement: Supplement 1 [file NIHPP2026.05.19.726350v1-supplement-1.pdf]

bioRxiv preprint doi: https://doi.org/10.64898/2026.05.19.726350; this version posted May 21, 2026. The copyright holder for this preprint (which was not certified by peer review) is the author/funder, who has granted bioRxiv a license to display the preprint in perpetuity. It is made available under a CC-BY 4.0 International license.

SUPPLEMENTAL FILES

The recreational-to-habitual shift in psychostimulant use is an economic demand parameter that is unrelated to drug consumption levels (under normal and punishment conditions).

Job et al

bioRxiv preprint doi: https://doi.org/10.64898/2026.05.19.726350; this version posted May 21, 2026. The copyright holder for this preprint (which was not certified by peer review) is the author/funder, who has granted bioRxiv a license to display the preprint in perpetuity. It is made available under a CC-BY 4.0 International license.

Supplemental Table Legends

Table S1: Variables obtained for the male (M) and female (F) subjects that self-administered methamphetamine (METH) and self-administration time demand curves could be fit using the exponential model. Note that n = 10 males were excluded and n = 0 females were excluded.

Table S2: Variables obtained for male subjects that self-administered sucrose and self-administration time demand curves could be fit using the exponential model. Note that n = 2 males were excluded.

Table S3: Variables obtained for female subjects that self-administered sucrose and self-administration time demand curves could be fit using the exponential model. Note that n = 2 females were excluded.

Supplemental Figure Legends

Figure S1. The demand curves of individuals (males and females) derived from saline self-administration time curve analysis using the new Behavioral Economic model for the Analysis of Self-administration Time-curve (BEAST). Each graph represents an individual. The n = 3 male rats that self-administered saline were labeled as rat75 – rat77 while the n = 10 female rats that self-administered saline were labeled as rat78-rat87. The R^2 for goodness of fit is written in each graph. Note that the exponential model for demand curves could not fit and/or yielded negative values for eValue and Pmax for all subjects. These individuals (the entire saline group) were excluded from further analysis.

Figure S2. Analysis of the distribution for all of the 10 variables suggests that males and females self-administering METH are not distinct groups. The criteria for confirming distinct

bioRxiv preprint doi: https://doi.org/10.64898/2026.05.19.726350; this version posted May 21, 2026. The copyright holder for this preprint (which was not certified by peer review) is the author/funder, who has granted bioRxiv a license to display the preprint in perpetuity. It is made available under a CC-BY 4.0 International license.

groups by biological sex is 1) there should be two clearly defined populations, and 2) these populations should mostly be non-overlapping. The graphs A-J show male versus female comparisons for all 10 variables. The histograms below the graphs A-J represent output from analysis of the distribution. The shaded and unshaded regions of the histograms represent females and males, respectively. The box and violin plots next to the histograms show the median value for the variables. There were significant differences between males and females only for slope (C) but not for all other variables (A-B, D-J). For all 10 variables, distribution analysis suggested the data represented a single normally-distributed population. By variable count, it may imply that males and females were different for 10% of the total number of variables analyzed but similar for 90% of the total number of variables analyzed. By percentage assessment males and females were more similar than they were different, overall.

Figure S3. Analysis of the distribution for each of the 10 variables suggests that males and females self-administering sucrose are not distinct groups. The criteria for confirming distinct groups by biological sex is 1) there should be two clearly defined populations, and 2) these populations should mostly be non-overlapping. The graphs A-J show male versus female comparisons for all 10 variables. The histograms below the graphs A-J represent output from analysis of the distribution. The shaded and unshaded regions of the histograms represent females and males, respectively. The box and violin plots next to the histograms show the median value for the variables. There were significant differences between males and females for MILD3, slope, R2H-shift rate, R2H-shift price (B, C, E and F) but not for all other variables (A, D, G-J). While different, MILD3, R2H-shift price and R2H-shift rate represented a single normally-distributed population (B, E and F). But even for slope (C), which revealed a bimodal distribution, the populations were overlapping, not clearly distinct, with each comprised of both males and females. By variable count, it may imply that males and females were different for 40% of the total number of variables analyzed but similar for 60% of the total number of variables analyzed. By percentage assessment males and females were more similar than they were different, overall.

bioRxiv preprint doi: https://doi.org/10.64898/2026.05.19.726350; this version posted May 21, 2026. The copyright holder for this preprint (which was not certified by peer review) is the author/funder, who has granted bioRxiv a license to display the preprint in perpetuity. It is made available under a CC-BY 4.0 International license.

Figure S4. Punishment-resistant versus Punishment-sensitive do not represent groups with distinct recreational-to-habitual shift rates for the drug: Open and close circles represent males and females, respectively. With the rationale that punishment-resistant (or shock-resistant, SR) and punishment-sensitive (or shock-sensitive, SS) are similar to high versus low takers under punishment conditions, we employed median split of their intake under punishment to separate the groups (see red highlighted graphs in G for METH and Q for sucrose). The other graphs are comparison of other variables of the same designated SR versus SS takers. For METH, SR versus SS takers (H) were (also) distinct for intake post-training (G) and intake post-punishment (I) – they were not distinct for intake-PRICE (J) or other intake variables (A-D) and R2H-shift variables (E-F). For sucrose, SR versus SS were distinct for intake post-training (K) and, except for slope, were distinct for all prior intake-type variables (intake-training, MILD3, Q_0, K,L and O). Note that sucrose SS and SR groups were not different for the R2H-shift price/rate (O- P). In summary, for METH and sucrose, SS and SR were distinct prior to the punishment phase (G, Q), but they were not different with regards to intake under the consumption-price curve (J, T) and they were not different with regards to R2H-shift variables (E-F, O-P).

Figure S5. High versus Low Takers do not represent groups with distinct recreational-to-habitual shift rates for the drug: Open and close circles represent males and females, respectively. The graphs A-J and K-T, respectively, represent comparison between high and low takers (HT and LT) for 10 variables for METH and sucrose, respectively. High and low takers were derived using median split of intake post-training (see red highlighted graphs in G for METH and Q for sucrose). The other graphs are comparison of other variables of the same designated high versus low takers. For METH, HT and LT (G) were distinct for most intake variables obtained during the training phase (MILD3 and Q_0 , B and D), and all intake after the training phase (H-J). HT and LT were not distinct with respect to R2H-shift price/rate (E-F). For sucrose, HT and LT (Q) were distinct for most intake variables obtained during the training phase (intake training, MILD3 and Q_0 , A-B and D), and intake during the punishment phase (R). HT and LT were not distinct with respect to R2H-shift price/rate (O-P). In summary, for METH and sucrose, HT and LT were not distinct with regards to R2H-shift price/rate (E-F, O-P).

bioRxiv preprint doi: https://doi.org/10.64898/2026.05.19.726350; this version posted May 21, 2026. The copyright holder for this preprint (which was not certified by peer review) is the author/funder, who has granted bioRxiv a license to display the preprint in perpetuity. It is made available under a CC-BY 4.0 International license.

Table S1

RatID	SEX	Intake training	MILD3	slope	Q_0	R2H-shift price	R2H-shift rate	Intake post-training	Intake-punishment	Intake post-punishment	Intake PRICE
1	M	458	16.67	0.32	24	2.963	4.135	52	6	8	0
5	M	970	66	4.32	56	3.487	3.853	11	0	14	0
6	M	756	47.33	1.35	43	3.846	4.341	303	36	241	21
7	M	587	62	4.02	80	7.341	6.823	410	37	296	36
8	M	1264	64.67	0.87	68	3.488	3.493	449	53	317	37
14	M	825	45.33	4.87	45	3.995	4.388	222	9	147	19
17	F	916	50.67	7.87	46	2.242	2.708	415	29	318	13
18	F	788	44.33	8.87	47	3.261	3.812	295	21	263	33
19	F	717	41.67	9.87	45	4.876	5.306	261	21	200	19
20	F	751	52	10.87	39	3.919	4.467	322	23	178	19
21	F	1105	63	11.87	64	4.999	5.042	407	32	260	28
22	F	905	59	12.87	55	5.023	5.252	288	21	163	16
23	F	872	61.33	13.87	58	5.137	5.404	366	36	228	33
24	F	775	48	14.87	40	2.745	3.256	358	36	224	24
25	F	505	50.33	15.87	36	5.284	9.382	310	37	151	45
26	F	635	34.33	16.87	32	1.810	2.639	282	7	99	0
27	F	607	41.67	17.87	37	3.633	4.219	185	32	169	1
28	F	946	63.33	18.87	53	3.819	4.143	358	25	229	22
29	F	986	52.67	19.87	56	4.725	4.899	455	39	215	0
30	F	317	22.33	20.87	24	4.421	5.645	156	0	40	3

bioRxiv preprint doi: https://doi.org/10.64898/2026.05.19.726350; this version posted May 21, 2026. The copyright holder for this preprint (which was not certified by peer review) is the author/funder, who has granted bioRxiv a license to display the preprint in perpetuity. It is made available under a CC-BY 4.0 International license.

Table S2

RatID	SEX	Intake training	MILD3	slope	Q_0	R2H-shift price	R2H-shift rate	Intake post-training	Intake-punishment	Intake post-punishment	Intake PRICE
31	M	5102	295.67	2.99	260	4.083	3.109	1915	150	494	67
32	M	7000	428.67	9.13	394	6.453	4.676	3203	145	793	83
34	M	3646	216.67	3.73	219	7.016	5.820	1525	87	672	33
35	M	3699	209.33	3.92	209	5.180	4.123	1336	105	678	52
36	M	3827	187.00	0.56	58	2.888	3.414	1253	79	867	38
37	M	2816	159.00	3.86	157	4.973	4.153	1099	58	458	63
38	M	3949	250.00	14.40	228	6.210	5.075	1857	85	470	66
39	M	5057	291.00	10.22	292	5.860	4.477	1910	85	237	33
40	M	4339	219.33	0.49	224	4.290	3.466	1389	93	705	38
41	M	4364	230.00	2.17	227	4.278	3.440	1453	99	112	23
42	M	5433	332.00	5.12	282	4.343	2.996	2122	115	343	92
44	M	2733	144.00	1.99	146	4.115	3.403	1048	83	574	43
45	M	3255	162.00	0.78	170	3.886	3.045	1025	28	507	6
46	M	2680	153.67	6.40	160	6.028	5.247	1062	69	660	34
47	M	1507	241.33	14.98	222	7.521	6.520	1162	80	856	209
48	M	4381	310.33	11.15	254	5.837	4.628	1336	220	700	129
49	M	3120	208.33	8.31	191	5.336	4.465	835	72	757	178
50	M	1811	163.67	9.43	158	6.638	5.957	803	82	453	116
51	M	4534	370.00	17.48	286	6.145	4.871	1602	175	1204	268
52	M	4316	251.67	6.71	235	4.903	3.738	1340	97	401	85

bioRxiv preprint doi: https://doi.org/10.64898/2026.05.19.726350; this version posted May 21, 2026. The copyright holder for this preprint (which was not certified by peer review) is the author/funder, who has granted bioRxiv a license to display the preprint in perpetuity. It is made available under a CC-BY 4.0 International license.

Table S3

RatID	SEX	Intake training	MILD3	slope	Q_0	R2H-shift price	R2H-shift rate	Intake post-training	Intake-punishment	Intake post-punishment	Intake PRICE
53	F	3508	188.00	2.77	179	3.378	2.485	1337	100	835	61
54	F	3972	241.00	4.01	202	3.579	2.565	1777	103	429	68
55	F	3318	135.33	-0.10	169	3.400	2.565	1189	65	749	70
57	F	2343	140.67	4.98	139	5.245	4.605	986	51	376	56
58	F	2705	161.00	4.87	146	4.450	3.708	1134	94	635	71
59	F	3848	189.00	1.25	195	3.463	2.485	1333	79	350	65
60	F	4966	241.67	1.40	256	4.472	3.924	1752	65	888	60
61	F	4865	268.33	2.70	255	4.382	3.136	1769	104	375	107
62	F	4152	202.00	1.66	214	3.848	2.694	1306	71	839	29
63	F	3665	218.33	2.18	184	3.599	3.381	1434	86	812	72
64	F	3350	171.00	0.94	172	4.074	3.924	1071	92	621	37
65	F	4070	233.67	1.73	209	3.831	2.838	1600	98	1118	63
66	F	2246	119.33	2.66	126	4.701	4.159	853	64	452	5
67	F	4400	224.00	1.68	223	3.833	3.423	1510	110	1045	64
68	F	5196	293.33	5.18	272	4.533	3.515	1862	133	1247	47
69	F	3048	220.33	8.56	178	5.376	4.489	804	101	704	189
70	F	3179	217.67	10.43	220	6.353	5.309	1070	105	994	280
71	F	1696	117.67	3.19	94	4.099	3.850	468	49	483	99
73	F	1579	90.00	0.19	79	2.675	3.303	406	59	264	63
74	F	1722	90.67	0.65	89	3.139	2.944	507	54	405	89

bioRxiv preprint doi: https://doi.org/10.64898/2026.05.19.726350; this version posted May 21, 2026. The copyright holder for this preprint (which was not certified by peer review) is the author/funder, who has granted bioRxiv a license to display the preprint in perpetuity. It is made available under a CC-BY 4.0 International license.

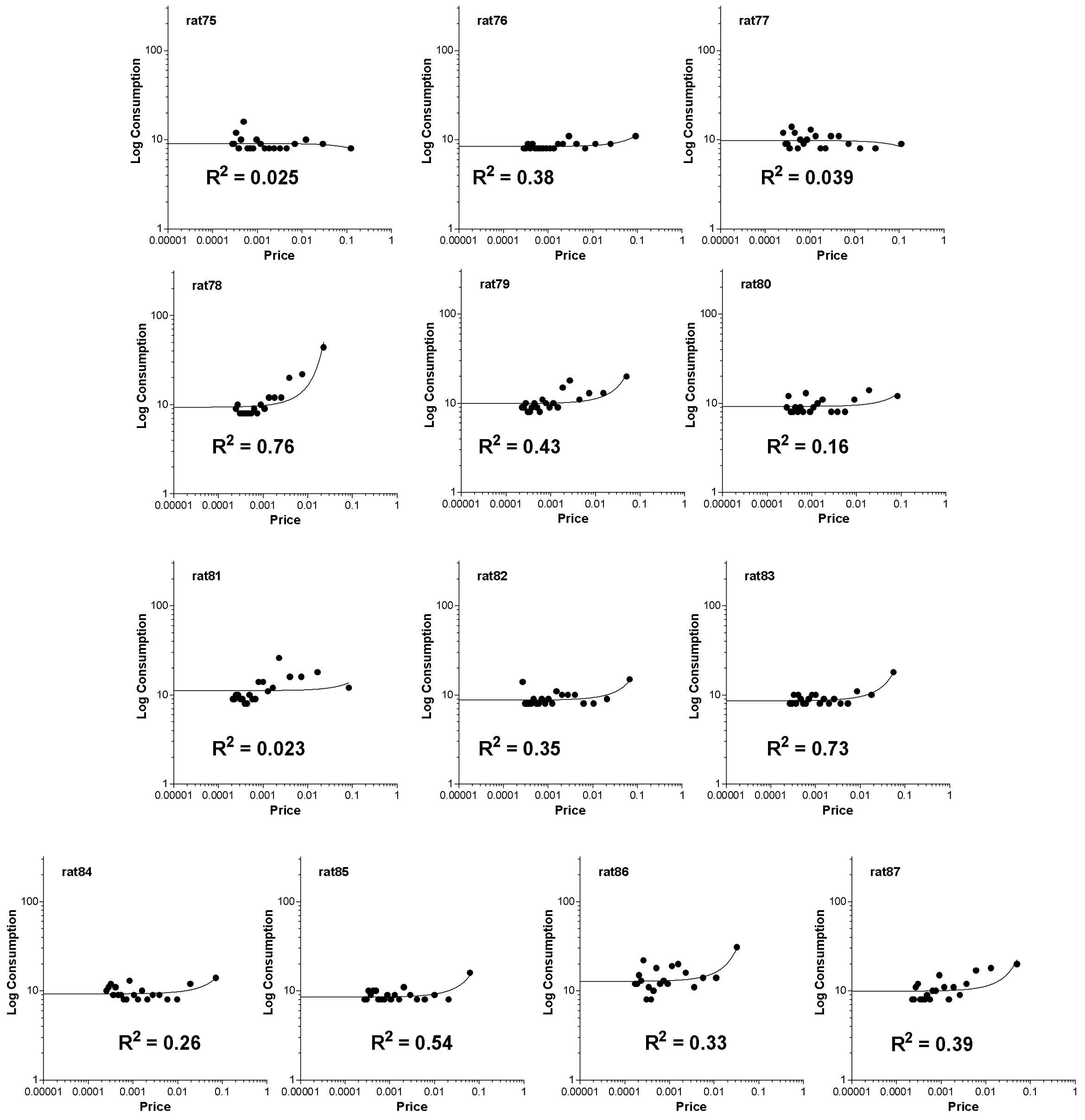

Figure S1

bioRxiv preprint doi: https://doi.org/10.64898/2026.05.19.726350; this version posted May 21, 2026. The copyright holder for this preprint (which was not certified by peer review) is the author/funder, who has granted bioRxiv a license to display the preprint in perpetuity. It is made available under a CC-BY 4.0 International license.

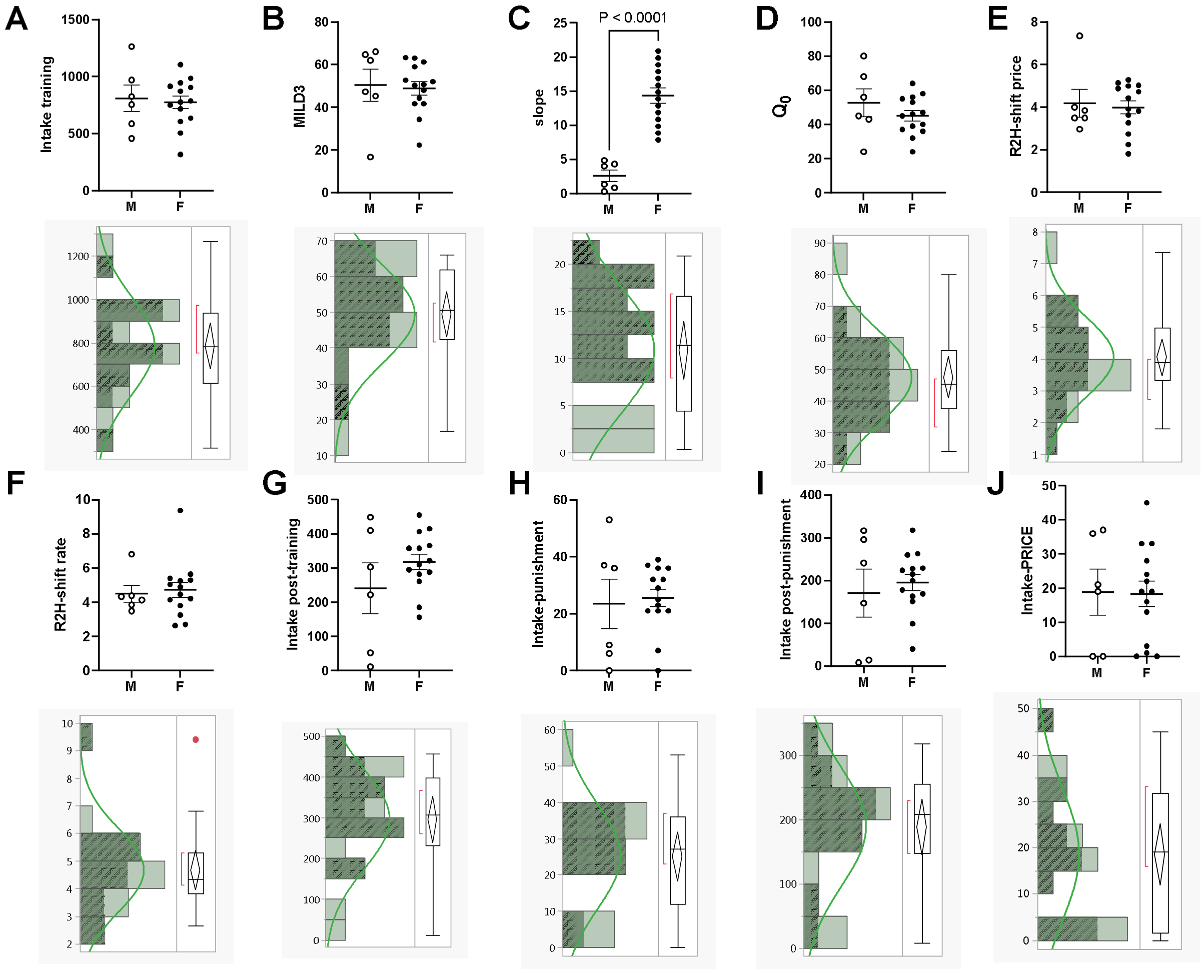

Figure S2

bioRxiv preprint doi: https://doi.org/10.64898/2026.05.19.726350; this version posted May 21, 2026. The copyright holder for this preprint (which was not certified by peer review) is the author/funder, who has granted bioRxiv a license to display the preprint in perpetuity. It is made available under a CC-BY 4.0 International license.

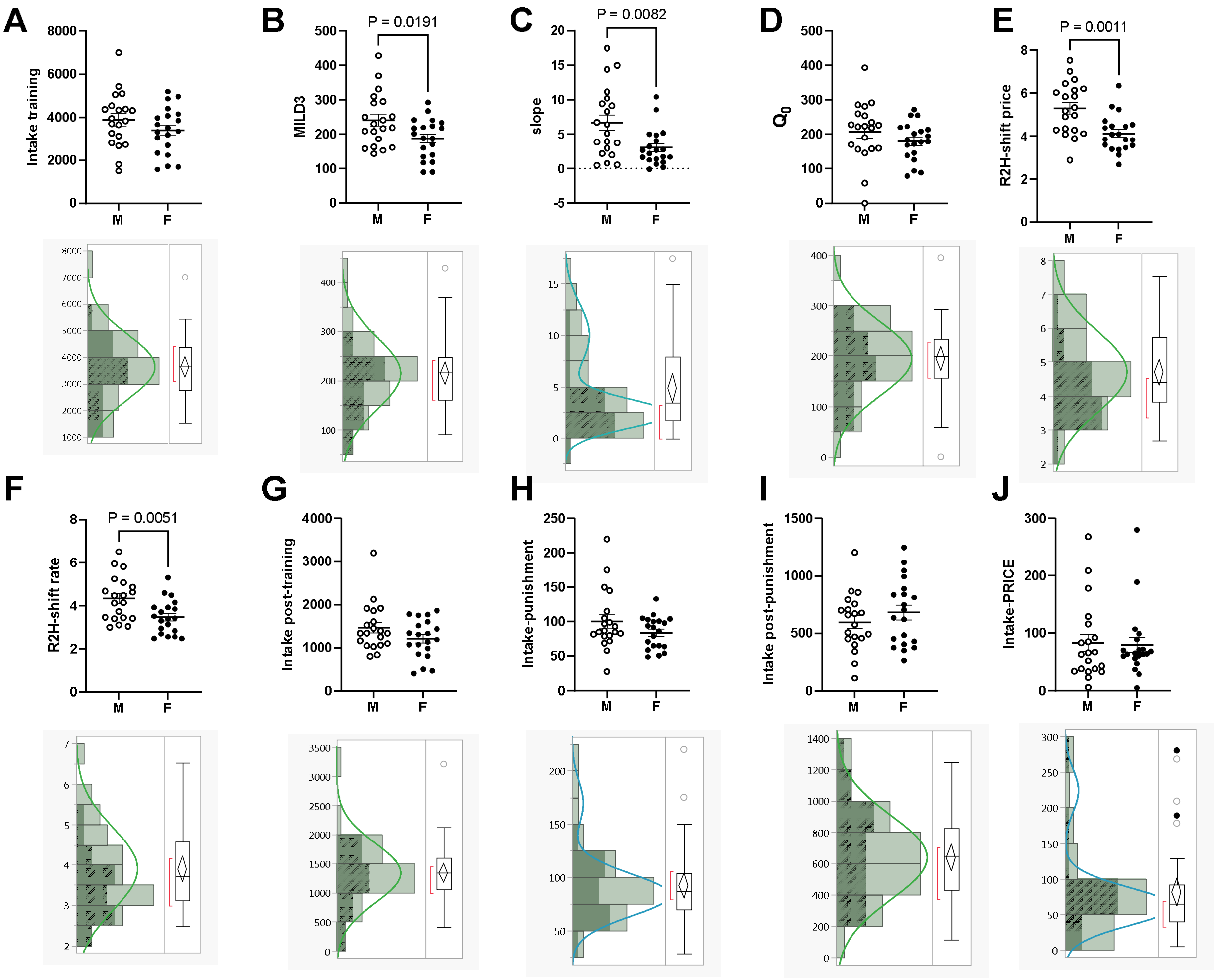

Figure S3

bioRxiv preprint doi: https://doi.org/10.64898/2026.05.19.726350; this version posted May 21, 2026. The copyright holder for this preprint (which was not certified by peer review) is the author/funder, who has granted bioRxiv a license to display the preprint in perpetuity. It is made available under a CC-BY 4.0 International license.

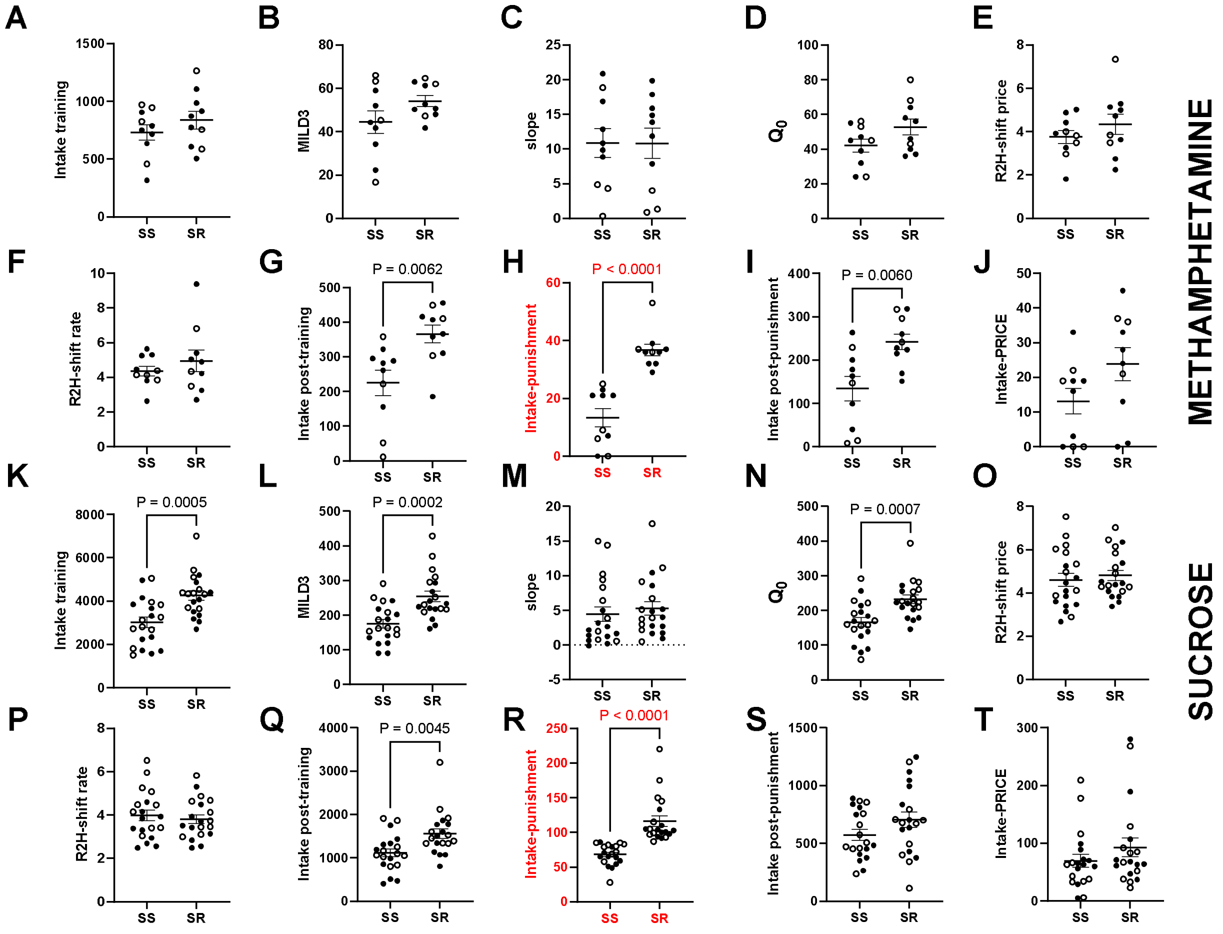

Figure S4

bioRxiv preprint doi: https://doi.org/10.64898/2026.05.19.726350; this version posted May 21, 2026. The copyright holder for this preprint (which was not certified by peer review) is the author/funder, who has granted bioRxiv a license to display the preprint in perpetuity. It is made available under a CC-BY 4.0 International license.

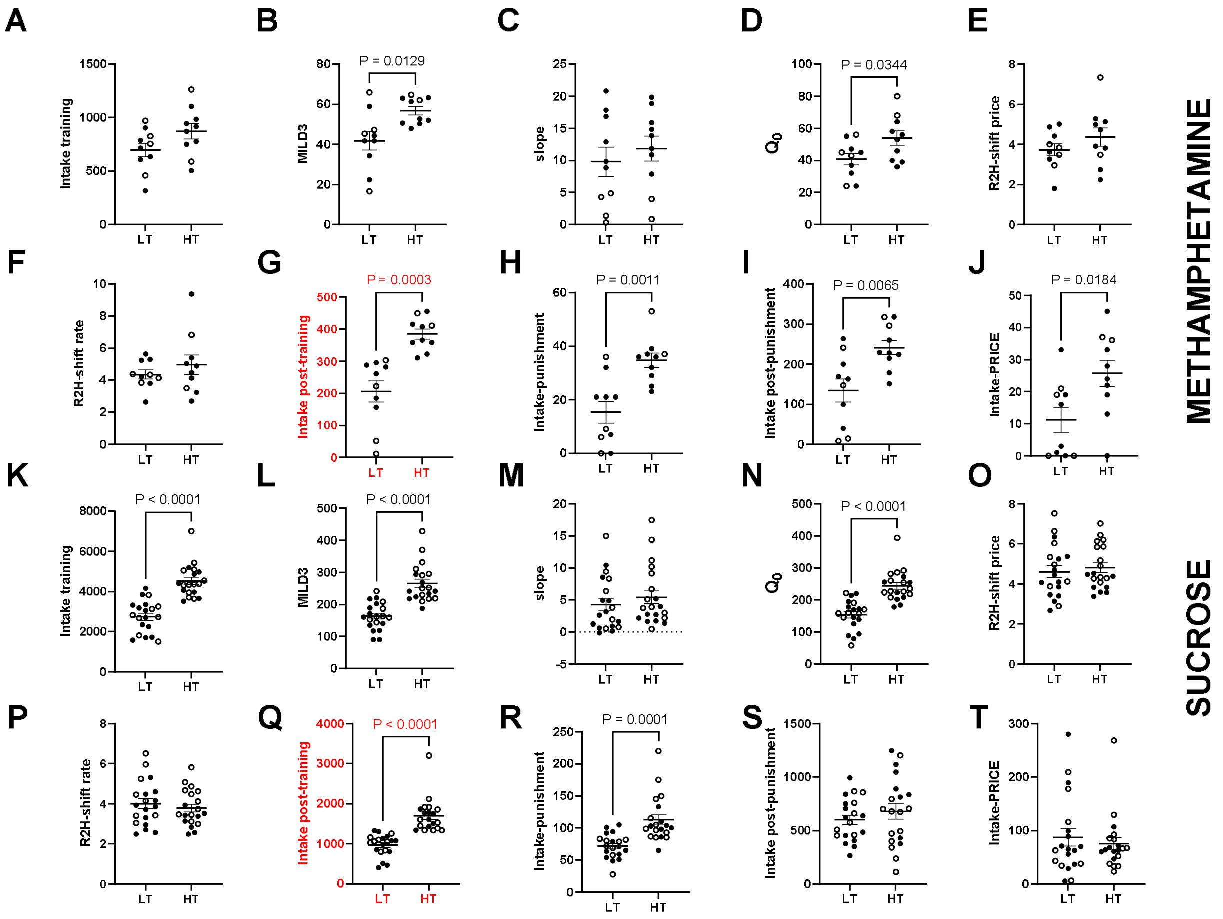

Figure S5

bioRxiv preprint doi: https://doi.org/10.64898/2026.05.19.726350; this version posted May 21, 2026. The copyright holder for this preprint (which was not certified by peer review) is the author/funder, who has granted bioRxiv a license to display the preprint in perpetuity. It is made available under a CC-BY 4.0 International license.
